# Research note: Gentle versus severe feather pecking: effects of synbiotic feed supplementation under chronic stress in laying hens

**DOI:** 10.1016/j.psj.2026.107055

**Published:** 2026-05-01

**Authors:** Nienke van Staaveren, Emily M. Leishman, Gillian Hughes, Anna Lea Nicklas, Paul Forsythe, Alexandra Harlander

**Affiliations:** a Department of Animal Biosciences, Campbell Center for the Study of Animal Welfare, University of Guelph, Guelph, Ontario, Canada; b Department of Animal and Veterinary Sciences, Aarhus University, Tjele, Denmark; c Faculty of Medicine & Dentistry, Department of Medicine, University of Alberta, Edmonton, AB, Canada

**Keywords:** Probiotics, Prebiotics, Fructooligosaccharides, Social buffering, Stress resilience

## Abstract

Feather pecking in laying hens is a complex behavior influenced by stress and the gut microbiota, with severe feather pecking (SFP) posing a major welfare concern and gentle feather pecking (GFP) representing a non-injurious, socially mediated behavior. This study investigated the effects of a commercial synbiotic supplement on SFP, GFP, and feather damage (FD) in hens exposed to chronic social and physical stressors. A total of 394 White Leghorn hens from high, low, and unselected feather pecking lines were housed in twelve floor pens. Pens received either synbiotic-supplemented or control feed, and within each feed treatment half of the pens were exposed to stressors including social mixing, perch removal, and litter reduction over a four-week period.

Behavioral observations and plumage assessments were conducted before and after stress exposure. Synbiotic supplementation did not significantly affect SFP or FD scores. However, stressors alone doubled SFP rates and tripled the likelihood of severe FD. GFP was significantly influenced by an interaction between feed and stress treatments; however post hoc comparisons indicated a trend toward higher GFP in stressed synbiotic-fed hens compared with their non-stressed counterparts, with no differences observed in control birds. These findings indicate that synbiotic supplementation via feed did not mitigate stress-induced SFP or FD, while subtle differences in GFP expression highlight the sensitivity of non-injurious pecking behaviors to behavioral context under stress. Overall, the results emphasize the importance of considering social and environmental context when evaluating microbiota-targeted interventions for laying hen behavior.

## Introduction

Domestic laying hens are social, group-living animals whose behavior is closely linked to the microbiome. Understanding this relationship may help to correct problematic behavior and improve welfare.

Exposure to chronic physical and psychosocial stressors in commercial settings influences hen behavior, notably pecking (Rodenburg et al., 2004). Severe feather pecking (SFP), a stress-related behavior, is characterized by forceful pecks that damage plumage, and often result in bald patches. SFP is distinct from aggressive pecking, which differs in motivation and physiological underpinning and represents a significant welfare concern across all housing systems (Rodenburg et al., 2004). Hens genetically selected for high SFP activity show an elevated heart rate and reduced parasympathetic tone during stress, indicating heightened autonomic reactivity (Kjaer and Joergenson, 2011). These findings suggest that SFP may reflect an exaggerated stress response, where increased arousal and reduced regulatory control contribute to the expression of the behavior. In contrast, alternative stress responses such as affiliative behavior may promote individual and group well-being. In birds, gentle feather pecking (GFP) may serve as such a prosocial or exploratory behavior by contributing to social recognition and group cohesion (Riedstra and Groothuis, 2002).

The gut-brain axis has recently emerged as a key regulatory pathway influencing behavior across species. Stress‑induced disruptions of this bidirectional system can alter gut microbiota composition, intestinal permeability, and neuroimmune signaling (Bharwani et al., 2016). In laying hens, it has been demonstrated that feather ingestion alters gut microbial composition and that SFP hens exhibit distinct cecal microbiome profiles, including reduced *Lactobacillus* abundance (Meyer et al., 2012; Huang et al., 2023). Importantly, in mammals, ingesting beneficial bacteria, such as *Lactobacillus* or *Bifidobacteria*, can modulate stress responses via neuroendocrine and neuroimmune pathways (Bharwani et al., 2016). Similarly, single-strain supplementation with *Lactobacillus rhamnosus* JB-1 reduced stress-induced SFP (Mindus et al., 2021). Taken together, these findings highlight that nutritional interventions containing *Lactobacillus* may allow for beneficial behavioral modifications in laying hens.

This study investigates the effects of a commercial synbiotic containing *Lactobacillus* administered through feed on feather-pecking behavior and plumage damage in laying hens. Specifically, it examines impacts on SFP, GFP, and plumage condition under exposure to unpredictable chronic stressors designed to simulate commercial farming environments.

## Materials and methods

### Animals and housing

All procedures were approved by the University of Guelph Animal Care Committee (AUP #4113). Three hundred and ninety‑four non‑beak‑trimmed White Leghorn laying hens were wing‑tagged at hatch and housed in 12 identical floor pens at the University of Guelph Poultry Research Station (Guelph, ON, Canada). Birds originated from high feather pecking (HFP), low feather pecking (LFP), and unselected control (UC) genetic lines (Kjaer et al., 2001).

At 15 weeks of age (woa), hens were placed into floor pens (31 ± 2 birds/pen; 4.7 m²) containing wood‑shaving litter, one suspended round feeder, a 5‑nipple drinker, three nest boxes on an elevated platform, and two perches (55 cm and 125 cm off the ground, 155 cm in length). Genetic lines were housed separately until 35 woa, after which HFP, LFP, and UC birds were mixed (∼ 10 per line per pen). Pens allowed auditory and olfactory, but not visual, contact. Lighting was provided at 20 lux from 05:00 to 19:00, with ad libitum access to feed and water.

### Synbiotic supplementation

Hens received a coarse crumble corn–soybean‑based layer‑breeder diet (2,886 kcal/kg ME; 18% CP; 6% fat). After a baseline period (43–44 woa), pens were assigned to either control feed or feed supplemented with a commercial synbiotic (45–48 woa; n = 6 pens/treatment). Researchers were blinded to feed assignment. The synbiotic was added at 0.5 kg/ton feed (0.5 kg per 1,000 kg) in the feed mill, providing 108 CFU/kg. It consisted of *Lactobacillus reuteri, Lactobacillus salivarius, Enterococcus faecium, Bifidobacterium animalis, Pediococcus acidilactici*, and fructooligosaccharides. Feed composition and recovery of *Lactobacillus* spp. and *Bifidobacterium* spp. in the feed were confirmed by SGS Agri‑Food Laboratories (Guelph, Canada).

### Chronic, unpredictable stress treatments

At 45 woa, three pens within each feed treatment were exposed to unpredictable chronic stressors for four weeks (stressed; n = 6 pens), while the remaining pens were left undisturbed (non‑stressed; n = 6 pens). Stressors included 10 rounds of social mixing, long‑term perch removal with brief 10‑min reintroduction periods, and removal of 50% (30 L) of the litter substrate. For social mixing, hens within each pen were divided into two subgroups (approximately 15 hens each), and each subgroup was transferred at 10:00 h to an identical pen containing an unfamiliar subgroup from another stressed pen within the same feed treatment (synbiotic or control). Perches were removed at the onset of the stress period and reintroduced briefly for 10 min on eight occasions, randomly distributed across the four‑week period to maintain unpredictability. Litter removal was carried out volumetrically using a container of known volume, with 30 L removed per pen from 45 woa onward.

### Behavioral observations and physical examination

Overhead cameras (Samsung SNO-5080R, IR, Samsung Techwin CO., Gyeongi-do Korea) recorded all pens. Individual hens were identified using silicon backpacks (8 × 6 × 0.5 cm). Based on pilot observations, behavior was recorded using all‑occurrence sampling from 10‑min morning recordings: 5 days during baseline (43–44 woa; 50 min per pen in total) and 9 days during treatments (45–48 woa; 90 min per pen in total). SFP was defined as forceful pecks and/or pulling feathers or skin, while GFP were light pecks without feather removal, where a bout ended when no pecks occurred for more than 4 s. Observers were blinded to treatment. Feather damage was assessed before (44 woa) and after (49 woa) treatments on four body regions (head/neck, back/rump, belly, and tail) using a 0–2 scale (0 being complete feathering; 2 being severe damage with featherless areas >2.8 cm, equivalent to Canadian two-dollar coin) by a blinded assessor (Decina et al., 2019).

### Statistical analysis

SAS software (Studio version 3.8, SAS Institute, Cary NC) was used for all statistical analyses. Pecking frequency was expressed as pecks per bird per 10 min and averaged at the pen level. Mixed models evaluated effects of feed, stress, week, and interactions. Feather damage was analysed using a multinomial model adjusting for genetic line and baseline score. Pen served as a random effect. Significance was set at *P* < 0.05; tendencies at 0.05 ≤ *P* ≤ 0.10.

## Results & discussion

**Fig. 1:**
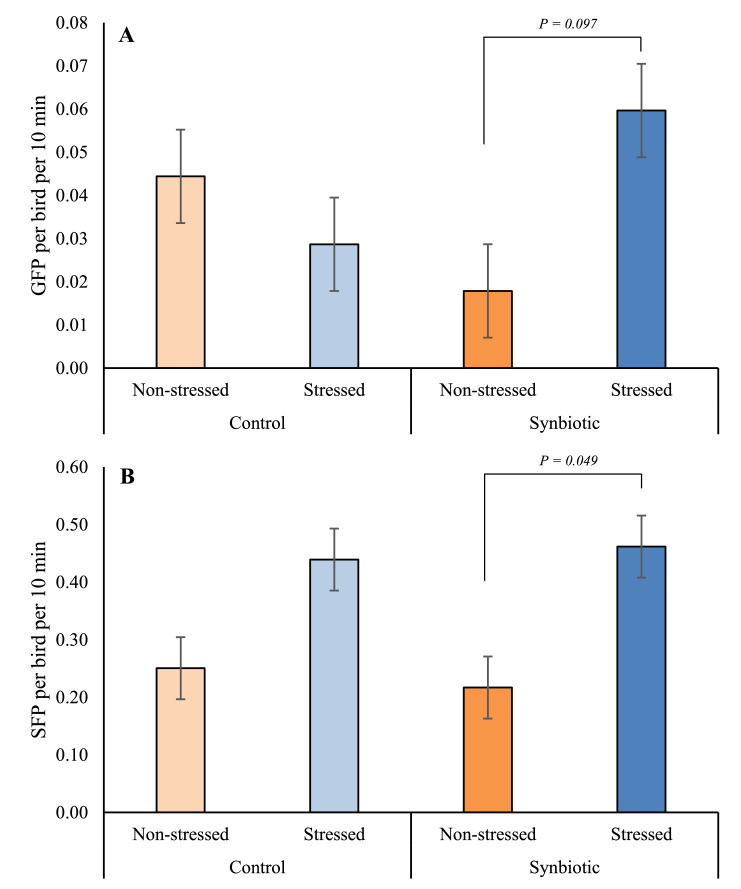
Least square (LS) means ± SE of the frequency of A) gentle feather pecks (GFP) and B) severe feather pecks (SFP). The number of GFP and SFP are expressed per bird per 10 min between 43 and 48 weeks of age in laying hens receiving commercial feed (Control) or commercial feed supplemented with a synbiotic (Synbiotic) under stressed (Stressed) and non-stressed (Non-stressed) conditions.

There was no significant interaction between feed type and stress treatments in determining SFP rates (F₁,₈ = 0.27, *P* = 0.6160, Fig. 1B), and synbiotic supplementation alone did not affect SFP. Birds receiving the synbiotic performed the same number of SFP bouts as control-fed birds (0.34 ± 0.038 vs. 0.34 ± 0.038 pecks/10 min/bird; F₁,₈ = 0.01, *P* = 0.9211). Feather damage (FD) outcomes were likewise unaffected by feed treatment (OR = 0.7, 95% CI: 0.34–1.28; Table 1). These results indicate that synbiotic supplementation via feed did not mitigate SFP behavior or associated plumage damage.

**Table 1:** Number and percentage of hens with no overall feather damage (score 0), moderate overall feather damage (score 1), or severe overall feather damage (score 2) at 49 weeks of age based on the stress treatment, feed treatment, or their interaction. Values within brackets represent row percentages.

Treatment Type	Feather damage		
	Score 0	Score 1	Score 2
*Stress treatment*			
Non-stressed	18 (9.73%)	30 (16.22%)	137 (74.05%)
Stressed	3 (1.62%)	20 (10.81%)	162 (87.57%)
*Feed treatment*			
Control	9 (4.81%)	29 (15.51%)	149 (79.68%)
Synbiotic	12 (6.56%)	21 (11.48%)	150 (81.97%)
*Interaction*			
Non-stressed / Control	6 (6.45%)	17 (18.28%)	70 (75.27%)
Non-stressed / Synbiotic	12 (13.04%)	13 (14.13%)	67 (72.83%)
Stressed / Control	3 (3.19%)	12 (12.77%)	79 (84.04%)
Stressed / Synbiotic	0 (0%)	8 (8.79%)	83 (91.21%)

In contrast, exposure to chronic social and physical stressors had a pronounced effect on SFP and FD. Stressed hens exhibited approximately twice the SFP rate of non-stressed hens (0.45 ± 0.038 vs 0.23 ± 0.038 pecks/10 min/bird; F₁,₈ = 16.19, *P* = 0.0038) and were nearly three times more likely to show severe FD (OR = 2.7, 95% CI: 1.38–5.32). Within the synbiotic treatment, stressed birds also performed more SFP than non-stressed birds (t₈ = −3.21, *P* = 0.0492; Fig. 1B), consistent with previous reports linking chronic stress exposure to increased SFP and plumage deterioration.

Gentle feather pecking (GFP) was significantly affected by an interaction between feed treatment and stress exposure (F₁,₈ = 7.07, *P* = 0.0289, Fig. 1A). However, pairwise comparisons within this interaction did not reach statistical significance only indicating a tendency for hens receiving the synbiotic to perform approximately three times more GFP than their non-stressed counterparts (t₈ = –2.73, *P* = 0.0973). No differences in GFP were observed between stressed and non-stressed birds receiving the control diet (t₈ = 1.03, *P* = 0.7386) and neither feed treatment alone (F₁,₈ = 0.04, *P* = 0.8425) nor stress alone (F₁,₈ = 1.45, *P* = 0.2632) significantly affected GFP. Thus, while the interaction term reflects a difference in the pattern of response between feed treatments, the non‑significant post hoc comparisons indicate that this effect should be interpreted as a trend rather than a robust treatment difference. The small sample size (n = 3) for the interaction term may have influenced the statistical significance of pairwise comparisons in this study.

The present study demonstrates that chronic social and physical stressors were the primary drivers of SFP and FD, whereas synbiotic supplementation delivered via feed did not mitigate these stress-related outcomes. The pronounced effects of stress on both SFP and FD are consistent with previous research showing that sustained environmental and social challenges increase SFP and plumage deterioration in laying hens (Rodenburg, et al., 2004). These results confirm the robustness of the applied stress model.

In contrast, synbiotic supplementation via feed did not affect SFP or FD severity, nor did it moderate the effects of stress on these measures. This suggests that, under the conditions of the present study, synbiotic supplementation delivered through feed was insufficient to reduce stress-induced SFP or FD. Notably, previous work using the same commercial synbiotic administered via drinking water reported that pecking rates remained stable during stress exposure, yet FD continued to worsen over time (Naim, et al., 2026). When considered alongside the present results, this suggests that preventing escalation of SFP alone may be insufficient to prevent progressive FD, particularly in birds already exhibiting substantial plumage loss at the onset of stress. Thus, SFP and FD may not respond in parallel to interventions targeting SFP behavior.

Gentle feather pecking (GFP) showed a significant feed x stress interaction; however, post hoc analyses revealed only a tendency toward increased GFP in stressed synbiotic-fed hens. This pattern should be interpreted cautiously and does not constitute evidence of a robust treatment effect. GFP has previously been described as a socially mediated or exploratory behavior rather than an injurious one (Riedstra and, Groothuis, 2002), and the present findings suggest that GFP may be more sensitive to contextual changes under stress than to feed supplementation per se. As such, GFP may provide complementary information on behavioral dynamics without directly reflecting welfare improvement.

Overall, these findings indicate that while synbiotic supplementation may influence behavioral patterns under certain conditions, its effectiveness in reducing stress-related SFP and FD appears limited when administered via feed to older hens with existing plumage damage. The results highlight the importance of considering stress load, baseline feather condition, and intervention context/mode when evaluating microbiota-targeted strategies for improving welfare in laying hens.

## CRediT authorship contribution statement

**Nienke van Staaveren:** Writing – review & editing, Writing – original draft, Visualization, Validation, Supervision, Project administration, Methodology, Investigation, Formal analysis, Data curation, Conceptualization. **Emily M. Leishman:** Writing – review & editing, Writing – original draft, Visualization, Validation, Project administration, Methodology, Investigation, Formal analysis, Data curation, Conceptualization. **Gillian Hughes:** Writing – review & editing, Writing – original draft, Investigation, Formal analysis, Data curation. **Anna Lea Nicklas:** Writing – review & editing, Writing – original draft, Visualization, Data curation. **Paul Forsythe:** Writing – review & editing, Writing – original draft, Project administration, Methodology, Funding acquisition, Conceptualization. **Alexandra Harlander:** Writing – review & editing, Writing – original draft, Visualization, Validation, Supervision, Resources, Project administration, Methodology, Investigation, Funding acquisition, Data curation, Conceptualization.

## Disclosures

The authors declare the following financial interests/personal relationships which may be considered as potential competing interests:

Alexandra Harlander reports equipment, drugs, or supplies was provided by Ontario Ministry of Agriculture Food and Rural Affairs. Alexandra Harlander reports article publishing charges and equipment, drugs, or supplies were provided by Natural Sciences and Engineering Research Council of Canada. Alexandra Harlander reports the synbiotic was provided by dsm-firmenich. If there are other authors, they declare that they have no known competing financial interests or personal relationships that could have appeared to influence the work reported in this paper.
